# A comparative absorption study of sucrosomial^®^ orodispersible vitamin D3 supplementation vs. a reference chewable tablet and soft gel capsule vitamin D3 in improving circulatory 25(OH)D levels in healthy adults with vitamin D deficiency—Results from a prospective randomized clinical trial

**DOI:** 10.3389/fnut.2023.1221685

**Published:** 2023-08-17

**Authors:** Aasiya Bano, Saida Abrar, Elisa Brilli, Germano Tarantino, Ali Akbar Bugti, Marco Fabbrini, Gabriele Conti, Silvia Turroni, Mahroo Bugti, Fauzia Afridi, Shah Mureed, Hakeem Zada, Ikram Din Ujjan, Saadia Ashraf, Aamir Ghafoor, Saeed Khan, Amjad Khan

**Affiliations:** ^1^PEOC, Department of Health, Balochistan, Quetta, Pakistan; ^2^Department of Obstetrics and Gynaecology, Lady Reading Hospital (LRH), Peshawar, Pakistan; ^3^Department of R&D, PharmaNutra S.p.A, Pisa, Italy; ^4^Department of General Surgery, Bolan Medical Complex Hospital (BMCH), Quetta, Pakistan; ^5^Unit of Microbiome Science and Biotechnology, Department of Pharmacy and Biotechnology, University of Bologna, Bologna, Italy; ^6^Department of Obstetrics and Gynaecology, BMCH, Quetta, Pakistan; ^7^Department of Obstetrics and Gynaecology, Khyber Teaching Hospital, Peshawar, Pakistan; ^8^Department of Paediatrics, BMCH, Quetta, Pakistan; ^9^Mubarak Diagnostic Laboratory and Research Center, Peshawar, Pakistan; ^10^Department of Pathology, Liaquat University of Medical and Health Sciences (LUMHS), Jamshoro, Pakistan; ^11^Department of Pulmonology, Khyber Teaching Hospital, Peshawar, Pakistan; ^12^Department of Gastroenterology, LRH, Peshawar, Pakistan; ^13^Department of Molecular Pathology, Dow University of Health Sciences, Karachi, Pakistan; ^14^Nuffield Division of Clinical Laboratory Sciences, Radcliffe Department of Medicine, University of Oxford, Oxford, United Kingdom; ^15^Department of Biochemistry, LUMHS, Jamshoro, Pakistan

**Keywords:** vitamin D deficiency, vitamin D3 supplementation, sucrosomial^®^ vitamin D3, orodispersible vitamin D3, vitamin D3 absorption

## Abstract

**Background:**

Vitamin D (Vit D) deficiency (VDD), associated with diverse health conditions, is commonly treated with Vit D3 supplements. However, the gastrointestinal (GI) absorption of Vit D3 in different formulations has not been well studied.

**Objective:**

We aimed to compare the absorption of an innovative phospholipids-sucrester matrix biodelivery vehicle-based (sucrosomial^®^) orodispersible Vit D3 preparation against a reference chewable tablet and soft gel capsule (SGC) Vit D3 formulations in Vit D-deficient healthy adults.

**Methods:**

In study 1, 25 subjects were randomized to receive a weekly single dose of 200,000 IU of sucrosomial^®^ Vit D3 (*n* = 12) or chewable tablet Vit D3 (*n* = 13) for 3 weeks. In study 2, 20 subjects were randomized to receive a single dose of 200,000 IU every other week of sucrosomial^®^ Vit D3 (*n* = 10) or SGC Vit D3 (*n* = 10) for 6 weeks. Circulatory 25-hydroxyvitamin D3 [25(OH)D] levels were reassessed after 2, 3, and 6 weeks in study 1 and after 4 and 6 weeks in study 2.

**Results:**

In study 1, after 2 weeks, circulatory 25(OH)D levels increased significantly in both Vit D3 treatment groups (*p* < 0.0001) but improved markedly in the sucrosomial^®^ Vit D3 group, with no further considerable change after 3 and 6 weeks in both groups. Overall, at all three follow-ups, sucrosomial^®^ Vit D3 treatment achieved significantly higher and sustained 25(OH)D levels (*p* < 0.001). In study 2, after 4 weeks, both Vit D3 treatment groups showed significant improvement in circulatory 25(OH)D levels (*p* < 0.0001) but substantially higher in the sucrosomial^®^ group with statistically significant differences between the two treatment groups (*p* = 0.02). At the 6-week follow-up, only subjects in the sucrosomial^®^ Vit D3 group showed a further increase in circulatory 25(OH)D levels (*p* = 0.049), but no further significant changes in the levels of the SGC Vit D3 group (*p* = 0.062), showing a statistically significant difference between the two treatment groups (*p* = 0.002). The Vit D3 treatment was well tolerated by all participants, and no treatment-emergent effects or serious adverse events were reported.

**Conclusion:**

Our results suggest that the sucrosomial^®^ Vit D3 preparation absorbs efficiently in the GI system, achieving adequately higher and sustained circulatory Vit D levels in VDD, and thus can effectively contribute to the body protection against VDD-associated health conditions.

**Clinical trial registration:**

clinicaltrials.gov, identifier: NCT05706259.

## 1. Introduction

**Figure 1:**
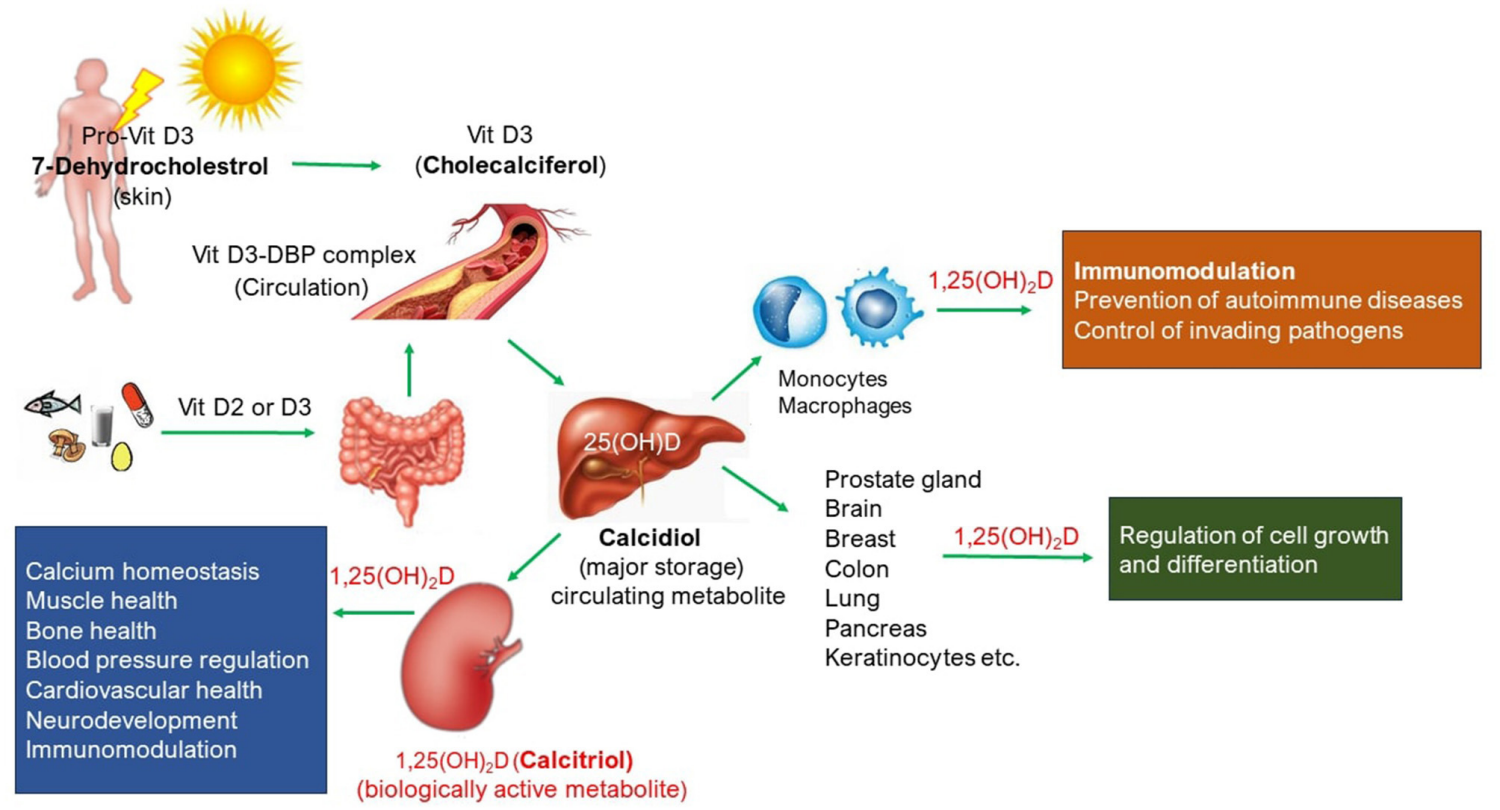
Schematic representation of circulatory Vit D and its physiological roles in the body.

Vitamin D (Vit D) is a steroid hormone that has a well-known physiological role in calcium homeostasis required for the growth and maintenance of healthy bones. Vit D absorbs calcium, magnesium, and phosphate from the gut into the circulatory system. A schematic representation of circulatory Vit D and its biological roles is shown in Figure 1. Most of the bioavailable Vit D is produced in the skin as Vit D3 (Cholecalciferol) from 7-dehydrocholesterol in response to sunlight exposure, and a small amount (~ 10%) is also absorbed from a healthy and balanced diet, both in D3 and D2 (ergocalciferol) forms. Further metabolism of Vit D to its major circulating form, 25-hydroxyvitamin D or 25(OH)D (calcidiol), and biologically active hormonal form, 1,25 dihydroxyvitamin D or 1,25(OH)_2_D (calcitriol), take place in the liver and kidney, respectively, but also in other tissues where 1,25(OH)_2_D produced serves as paracrine/autocrine function such as the skin, cells of the immune system, parathyroid gland, intestinal epithelium, prostate, breast, brain, colon, and pancreas. Dietary Vit D comes in both D_2_ and D_3_ forms, but they are metabolized to the same active form of Vit D the body needs, i.e. calcitriol. Vit D receptors are ubiquitously present in the body (1) and thus play a plethora of physiological roles (Figure 1). For instance, Vit D deficiency (VDD), defined as circulatory 25(OH)D levels < 20 ng/ml, is not only linked with rickets or osteomalacia or osteoporosis but also with the risk of cardiovascular diseases, autoimmune diseases (such as type 1 diabetes, rheumatoid arthritis, inflammatory bowel disease, and multiple sclerosis), impaired mental health conditions (such as depression, anxiety, and Parkinson's disease), tuberculosis, and cancer (breast cancer and colon cancer) (2). Vit D has also shown protective effects against respiratory tract infections (3–5), and there is emerging evidence of a possible association between VDD and progression to severe illness and mortality in patients with COVID-19 (6). In recent years, the role of Vit D in immune and inflammatory responses has been extensively studied. Reported evidence suggests Vit D as an endogenous regulator of both innate and adaptive immunity (3–5, 7–11). Circulatory Vit D levels affect the proliferation and differentiation of immune cells, and its deficiency results in impaired immune response and increases the risk of developing immune-related diseases.

The lack of exposure to sunlight, inadequate nutritional intake, malabsorption syndrome, and increased skin pigmentation have been identified as the main reasons for VDD (12). Vit D deficiency is a common metabolic/endocrine abnormality and a global public health problem. Approximately, over one billion people worldwide are Vit D deficient, and the deficiency is frequently undiagnosed. The prevalence of VDD in Europe, USA, and the Middle East ranges from 20 to 90% (13, 14), with similar trends also reported in Australia, India, Africa, South America, Turkey, and Lebanon (13–16). In South Asia, Pakistan has the highest rate of VDD of 53.5%, mostly in its female population (63%) (17).

Sunlight exposure and dietary intake alone are insufficient to maintain optimal circulatory Vit D levels (30–50 ng/ml) required for physiological functions (13, 18). To achieve and maintain adequate circulatory Vit D levels, over-the-counter Vit D supplements are often consumed, which can be taken daily, weekly, or monthly. Currently, there is no international consensus on the optimal dose of Vit D supplementation. Dosage recommendations vary across countries, ranging from 400 to 2000 IU Vit D per day (10–50 μg). Supplemental Vit D3 form is generally preferred because it appears to be more efficacious in raising circulatory 25(OH)D concentrations than the D2 form. Vit D3 supplement is usually administered orally and produced in various forms, such as oral drops, tablets, soft gel capsules (SGC), and injectable oily solutions. However, despite the widespread consumption of Vit D supplements for treating VDD, the absorption (and hence efficacy) of different Vit D formulations has not been well studied (19). Vit D, being a fat-soluble vitamin, is immiscible in the aqueous gut environment which presents challenges to its absorption. Its absorption in the intestinal mucosa is highly dependent on its food/delivery matrix dissolution in the gastrointestinal (GI) fluid (20–27) (stomach juices, liver bile, liver, and pancreatic secretions) and the integrity of the intestinal wall. The absorption is further limited by multiple factors including the physiochemical state of Vit D3 food/delivery matrix, Vit D interaction with other compounds in the food such as cholesterol, fatty acids, and dietary fibers, and factors associated with humans such as malabsorption syndrome, other chronic diseases, age, medication use, surgery, obesity, and genetic variation (28). To overcome these negative factors and intestinal barriers, the development of more bio-accessible delivery vehicles can enhance absorption and achieve adequately higher Vit D levels in the circulatory system for physiological roles.

The present study aimed to assess the GI absorption (hence efficacy) of a high-dose (200,000 IU) supplementation of an innovative oral phospholipids-sucrester matrix delivery vehicle-based, also known as sucrosomial^®^ (29) orodispersible Vit D3 formulation vs. a reference marketed chewable tablet and soft gel capsule (SGC) Vit D3 formulation in raising circulatory 25(OH)D levels, as well as safety, and tolerability in Vit D deficient but otherwise healthy adults. It is anticipated that the unique structural, physiochemical, and pharmacokinetic characteristics of the sucrosomial^®^ Vit D3 preparation could offer several advantages over the conventional oral Vit D3 formulations such as stability in the GI tract, efficient intestinal absorption, increased Vit D3 bioavailability, and improved patient compliance, particularly in pediatric and geriatric patients, and those with malabsorption syndrome.

## 2. Materials and methods

### 2.1. Study design and participants

This was a prospective, parallel group, open-label, randomized clinical study conducted at Bolan Medical Complex Hospital (BMCH), Quetta, and Lady Reading Hospital (LRH), Peshawar, Pakistan, from 1 February 2023 to 8 April 2023. This exploratory study assessed the absorption of a new phospholipids-sucrester matrix delivery vehicle-based (sucrosomial^®^) orodispersible Vit D3 (Surosomial^®^ UltraD3 Cholecalciferol 100,000 IU, Alesco srl, Pisa, Italy, patent number WO 2021/111404) supplement compared to a randomly selected reference (local market) chewable tablet (Admore^®^, Biolex, Cholecalciferol 100,000 IU, Pharma, PK) (study 1, BMCH) and SGC (Opt-D^®^, Cholecalciferol 200,000 IU, PharmEvo, PK) (study 2, LRH) Vit D3 supplements in healthy adults with VDD. The study was approved by the BMCH Ethical Board Committee (Ref. No. BMCH/EBC/5500) and LRH Institutional Review Board (Ref. No. 696/LRH/MTI) and was conducted in accordance with the guidelines of the Declaration of Helsinki and Good Clinical Practice. All participants provided informed written consent. The study was registered on clinicaltrials.gov, identifier number NCT05706259.

Inclusion criteria for the study were as follows: healthy male and female adults aged 18–45 years; VDD as shown by serum 25(OH)D levels < 20 ng/ml; body mass index (BMI) 18.9–29.9 kg/m^2^; vital signs (systolic blood pressure 100–139 mmHg, diastolic blood pressure 50–89 mmHg, and heart rate 50–90 bpm measured after 5 min at rest in the sitting position); willing to provide informed written consent; and able to cooperate with investigators and comply with study requirements.

Exclusion criteria were as follows: clinically significant abnormal laboratory parameters including hematology, calcium, liver enzymes, creatinine, ferritin, C-reactive protein (CRP), and D-dimer, indicative of physical illness, especially hypercalcemia and hypercalciuria; hypersensitivity to Vit D supplements; history of renal, hepatic, gastrointestinal, cardiovascular, respiratory, skin, hematological, endocrine, or neurological diseases; prior use of supplements containing calcium, Vit D, or magnesium, 4 weeks before the start of the study; participation in the evaluation of any investigational product or blood donation in the past 3 months; or any other significant disease or disorder that in the opinion of the treating physician may either put the participant at risk because of participation in the study or influence the results of the study or the participant's ability to participate in the study.

### 2.2. Subjects' enrolment, randomization, and Vit D3 treatment

**Figure 2:**
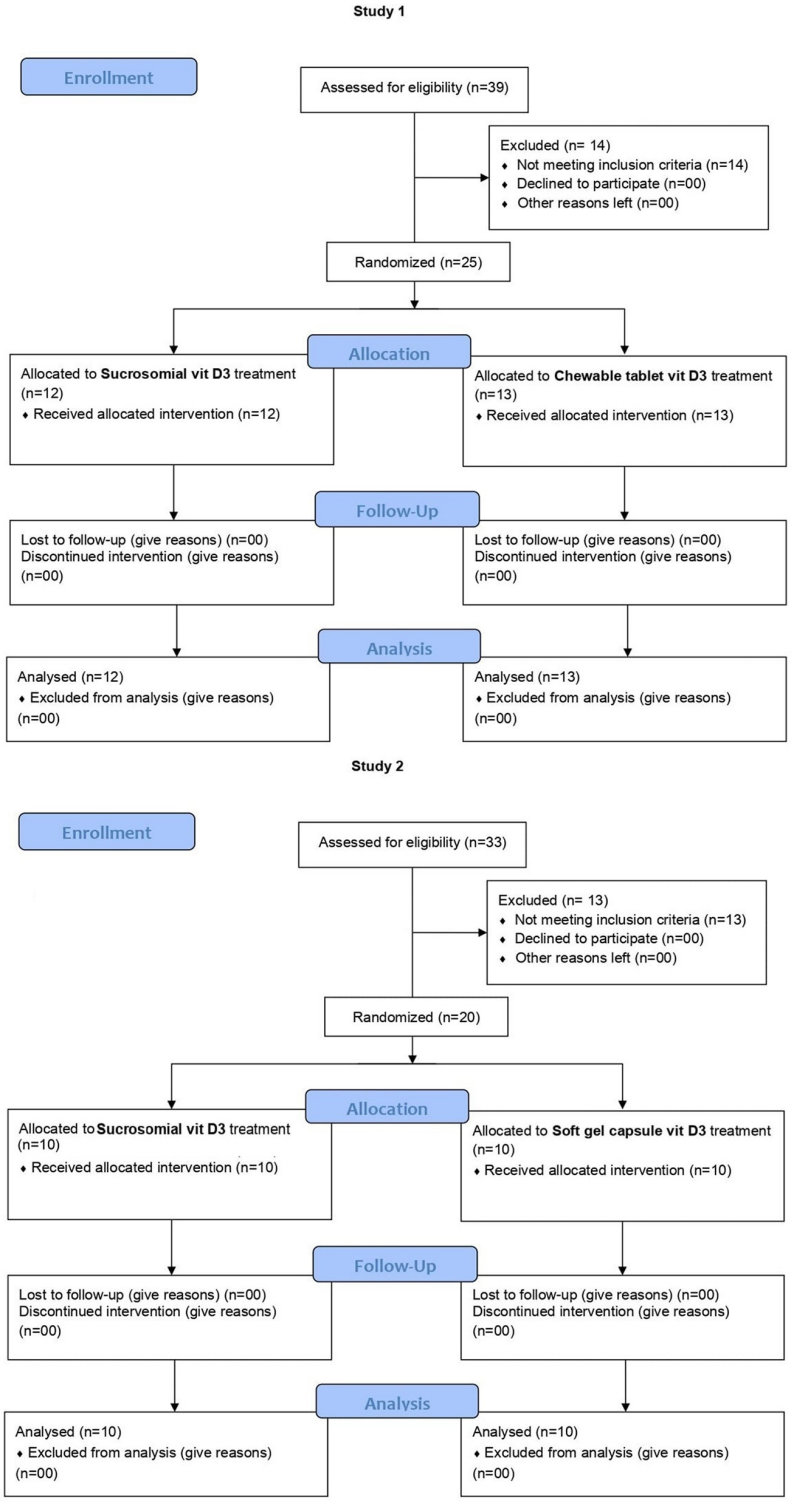
CONSORT flow diagram for studies 1 and 2.

The trial CONSORT flow diagram is shown in Figure 2. In study 1 (BMCH), subjects were randomly screened in the community for VDD. In total, 25 subjects, meeting the inclusion criteria and none of the exclusion ones, were enrolled by the clinical support team and were then allocated by an independent member of support staff who was not involved in the study, in a 1:1 ratio using computer-generated permuted block randomization sequence to receive the sucrosomial^®^ Vit D3 (Surosomial^®^ UltraD3 Cholecalciferol 100,000 IU) (*n* = 12) or the chewable tablet Vit D3 (Admore^®^, Biolex, Cholecalciferol 100,000 IU) (*n* = 13) supplement, at a single weekly dose of 200,000 IU (same day and same time) for 3 consecutive weeks. Treatment allocation was concealed from participants and study staff. Subjects were given the respective Vit D3 supplement by the treating physician to be taken at home the next morning after a light breakfast. Follow-up serum 25(OH)D levels were evaluated after 2, 3, and 6 weeks. Similarly, in study 2 (LRH), postgraduate trainee doctors working in the Department of Gynecology and Obstetrics were randomly screened for VDD. Following the inclusion/exclusion criteria, a total of 20 subjects were enrolled and randomized in a 1:1 ratio to receive either the sucrosomial^®^ Vit D3 (Surosomial^®^ UltraD3 Cholecalciferol 100,000 IU) (*n* = 10) or the SGC Vit D3 (Opt-D^®^, Cholecalciferol 200,000 IU) (*n* = 10) supplement, in a single dose of 200,000 IU every other week for 6 weeks. Follow-up serum 25(OH)D levels were evaluated after 4 and 6 weeks. In both studies, each participant received a total of 3 doses, each of 200,000 IU (total 600,000 IU) of the respective Vit D3 supplement over a period of 6 weeks. All participants were instructed to maintain their lifestyles including dietary habits and sun exposure throughout the study period, to minimize interference with daily routines. Subjects were advised to visit the designated independent clinical laboratory on the specified day for blood sample collection. All participants were instructed to note any adverse events during the entire 6-weeks study period. Safety parameters including biochemistry (serum calcium levels, liver function enzymes, and creatinine) and hematology were evaluated at the baseline (prior to Vit D3 supplementation) and at the final 25(OH)D evaluation.

### 2.3. Circulatory 25(OH)d levels evaluation

In both studies, blood sampling and evaluation of serum 25(OH)D levels and other laboratory parameters were performed by independent clinical diagnostic laboratories: in study 1 (BMCH), by Jinnah Sindh Medical University (JSMU) laboratory on Cobas e601 instrument and in study 2 (LRH), by Alkhidmat Diagnostic Laboratory on Cobas e411 instrument, both used the electrochemiluminescence (ECL) assay (Roche Diagnostics, Indianapolis, IN, USA) method.

### 2.4. Study endpoints

The primary endpoint of the study was to compare the absorption of sucrosomial^®^ Vit D3 vs. the chewable tablet Vit D3 (study 1) and SGC (study 2) Vit D3, measured as an increase in the serum 25(OH)D levels during the 6-weeks treatment period. The secondary endpoint was to assess the safety and tolerability of Vit D3 treatment in the form of any significant effect on any laboratory biochemistry including liver function enzymes, creatinine, and hematological parameters, evaluated before and after the completion of Vit D3 treatment.

### 2.5. Statistical analysis

To compare the absorption (effectiveness) of sucrosomial^®^ Vit D3 supplement vs. the chewable tablet Vit D3 (study 1) or SGC Vit D3 formulations (study 2) in raising the circulatory 25(OH)D levels, Wilcoxon tests were used. According to age, weight, and BMI, evenness was also tested, with Wilcoxon tests for age and BMI, and Pearson's chi-square test with Yates' continuity correction for gender distribution with respect to formulations. All *p*-values were adjusted for multiple comparisons with Benjamini–Hochberg correction (False Discovery Rate - FDR) (30). All consolidated values are reported as mean ± standard error of the mean (SEM). The Wilcoxon tests were used to assess the safety and tolerability of Vit D3 treatment in terms of significant effect on any of the blood biochemistry and hematological parameters. All statistical analyses were performed using R version 4.2.2 (31) and the R package “ggplot2” (32) for graphical representations. Based on previously reported studies (22, 26, 27, 33), a sample size of 20 subjects in each study was considered sufficient for this exploratory descriptive study.

## 3. Results

### 3.1. Baseline characteristics

The baseline demographics and clinical characteristics of subjects in the two studies are presented in Table 1. In study 1, overall, the mean age was 34.1 ± 1.8 years, baseline serum 25(OH)D level was 11.3 ± 0.7 ng/ml, and the cohort included 13 men and 12 women. There were more men in the sucrosomial^®^ Vit D3 group (*n* = 8), while more women (*n* = 8) in the chewable tablet Vit D3 treatment group. However, this apparent gender bias was not significant (*p* = 0.313). At the baseline, the two Vit D3 treatment groups were otherwise reasonably balanced in terms of age and clinical characteristics including body weight, BMI, circulatory 25(OH)D, calcium, and hemoglobin levels.

**Table 1:** Overall baseline demographic and clinical characteristics of the participants in the two studies.

**Characteristic**	**Study 1**		**Study 2**	
	**Sucrosomial**^®^ **Vit D3 treatment group (*****n*** = **12)**	**Chewable tablet Vit D3 treatment group (*****n*** = **13)**	**Sucrosomial**^®^ **Vit D3 treatment group (*****n*** = **10)**	**Soft gel capsule Vit D3 treatment group (*****n*** = **10)**
**Sex**				
Male, *n*	8	5	0	0
Female, *n*	4	8	10	10
Age (years)	31.3 ± 1.7	36.6 ± 3.0	27.5 ± 0.9	31.3 ± 1.8
Body weight (kg)	71.8 ± 3.0	64.3 ± 2.8	56.9 ± 4.4	62.7 ± 1.8
Body mass index (kg/m^2^)	24.1 ± 1.0	23.8 ± 0.8	24.3 ± 1.2	25.4 ± 1.0
Serum 25(OH)D levels (ng/ml)	9.6 ± 0.7	12.8 ± 1.0	11.6 ± 0.8	12.6 ± 0.9
Serum calcium levels (mg/dL)	9.9 ± 0.1	9.9 ± 0.1	9.5 ± 0.2	9.6 ± 0.2
Hemoglobin (g/dL)	15.8 ± 0.5	13.7 ± 0.7	12.3 ± 0.2	12.2 ± 0.3

Data are presented as mean ± SEM or numbers. No statistically significant difference was observed in any variable between the two Vit D3 treatment groups within each study.

In study 2, overall, the mean age was 29.4 ± 1.1 years, all were women, and the baseline mean serum 25(OH)D level was 12.1 ± 0.6 ng/ml. At the baseline, the two treatment groups were balanced in terms of participants' demographic and clinical characteristics.

In both studies, treatment adherence was 100% as reported by all participants on the dosage day through a phone call. The sucrosomial^®^ Vit D3 was taken by dissolving in the mouth either without water or with a sip of water. In both studies, all participants completed the study, and there were no dropouts.

### 3.2. Effect of Vit D3 supplementation

#### 3.2.1. Study 1

**Figure 3:**
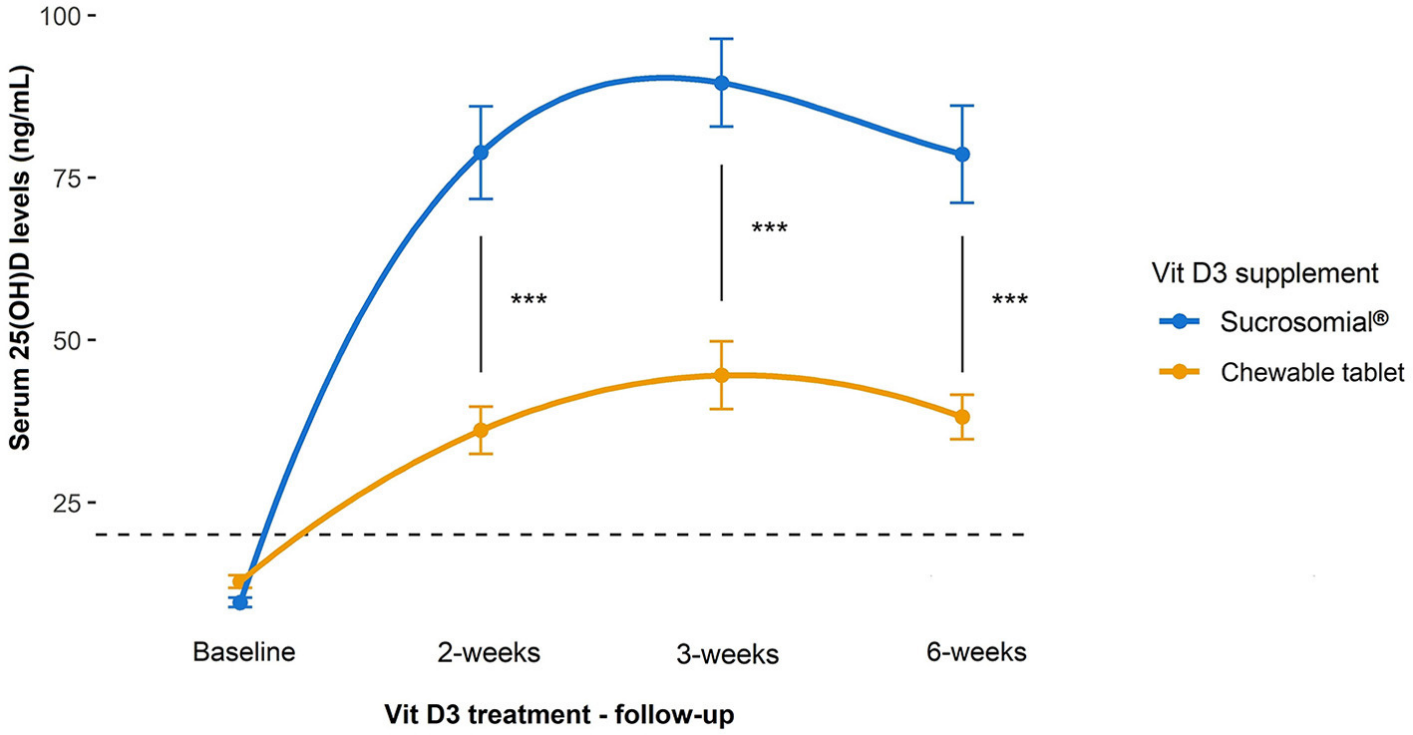
Efficacy of sucrosomial^®^ orodispersible Vit D3 vs. chewable tablet Vit D3 supplementation on serum 25(OH)D levels in Vit D-deficient healthy adults. Graphs showing serum 25(OH)D levels (expressed as mean ± SEM) measured before and after Vit D3 treatment (a single weekly dose of 200,000 IU for 3 consecutive weeks) in Vit D-deficient healthy adults. Sucrosomial^®^ Vit D3, *n* = 12; chewable tablet Vit D3, *n* = 13. The dashed horizontal line shows the serum Vit D deficiency threshold [25(OH)D < 20 ng/ml]. Wilcoxon test was used to assess the significance of changes in circulatory 25(OH)D levels over time within each Vit D3 treatment group and between groups. To simplify the data presentation, only significant inter-group differences are shown (for intra-group differences over time, please refer to the main text). ^***^p < 0.001.

Follow-up serum 25(OH)D levels measured during Vit D3 treatment in both groups are shown in Figure 3. After 2 weeks of Vit D3 treatment, both groups showed a significant increase in the serum 25(OH)D levels (*p* < 0.0001) but rose substantially higher in the sucrosomial^®^ Vit D3 group, with a statistically significant difference between the two groups (*p* = 0.0003), i.e. sucrosomial^®^ Vit D3: from baseline mean ± SEM 9.6 ± 0.7 to 78.8 ± 7.1 ng/ml vs. chewable tablet Vit D3: from baseline 12.8 ± 1.0 to 36.1 ± 3.6 ng/ml. After 3 weeks, both treatment groups showed a further small and comparable increase in serum 25(OH)D levels to 89.6 ± 6.7 ng/ml and 44.6 ± 5.2 ng/ml, in the sucrosomial^®^ Vit D3 and chewable tablet Vit D3 supplement groups, respectively. After 6 weeks, serum 25(OH)D levels showed a small and comparable decrease to 78.6 ± 7.5 ng/ml and 38.1 ± 3.4 ng/ml, in the sucrosomial^®^ Vit D3 and chewable tablet Vit D3 groups, respectively. At all time points, sucrosomial^®^ Vit D3 achieved significantly higher and sustained circulatory 25(OH)D levels as compared with the chewable tablet Vit D3 (*p* < 0.001) supplement. These results suggest an efficient absorption of the sucrosomial^®^ Vit D3 preparation as compared to the chewable tablet Vit D3 supplement.

#### 3.2.2. Study 2

**Figure 4:**
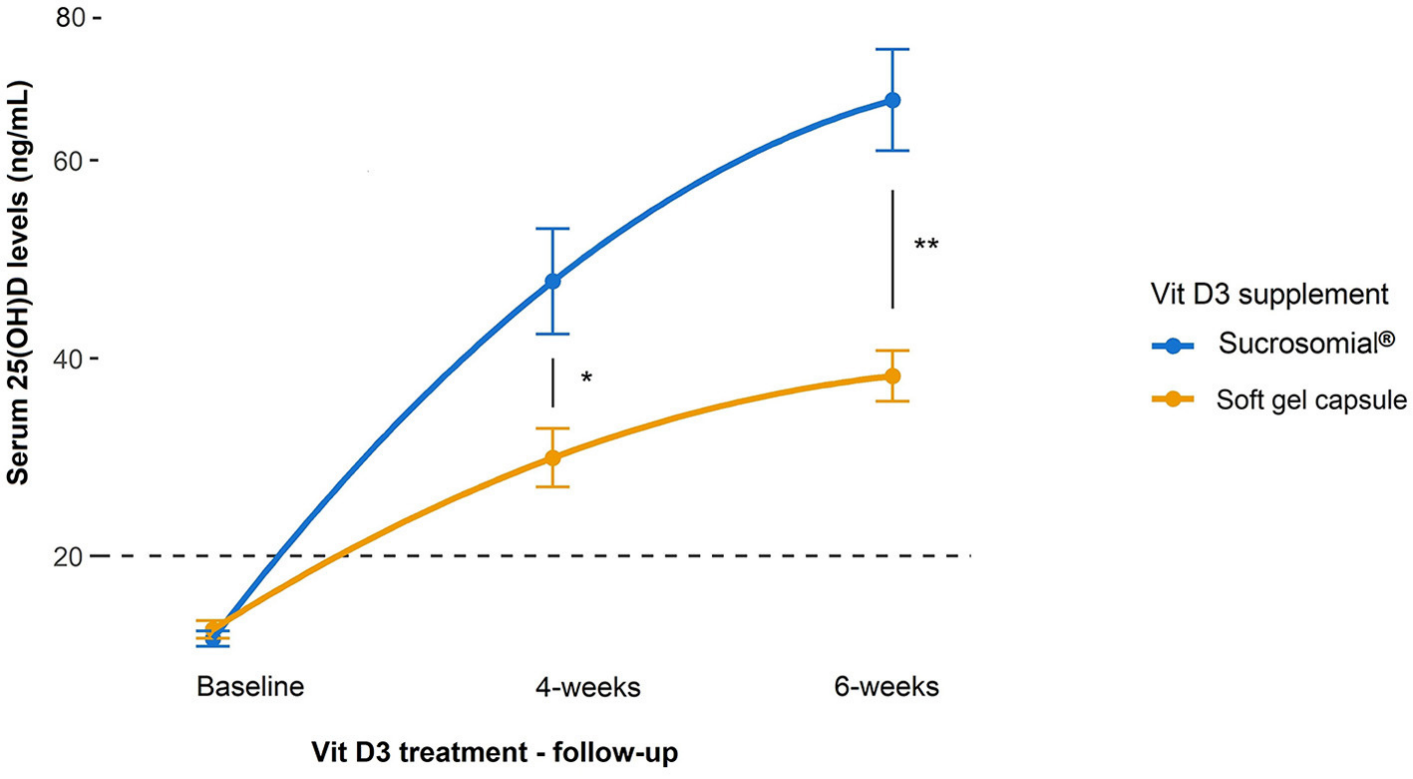
Efficacy of sucrosomial^®^ orodispersible Vit D3 vs. soft gel capsule (SGC) Vit D3 supplementation on serum 25(OH)D levels in Vit D-deficient healthy adults. Graphs showing serum 25(OH)D levels (expressed as mean ± SEM) before and after Vit D3 treatment (single weekly dose of 200,000 IU every other week for 6 weeks) in Vit D-deficient healthy female adults. Sucrosomial^®^ Vit D3, *n* = 10; soft gel capsule Vit D3, *n* = 10. The dashed horizontal line shows the serum Vit D deficiency threshold (20 ng/ml). Wilcoxon test was used to assess the significance of changes in circulating 25(OH)D levels over time within each Vit D3 treatment group and between groups. To simplify the data presentation, only significant inter-group differences are shown (for intra-group differences over time, please refer to the main text). ^*^p < 0.05, ^**^p < 0.01.

In study 2, like study 1, sucrosomial^®^ Vit D3 supplementation led to a substantial increase in follow-up serum 25(OH)D levels as compared to the SGC Vit D3 supplement, which showed only a mild increase (Figure 4). Although after 4 weeks follow-up, serum 25(OH)D levels increased significantly from the baseline in both treatment groups (*p* < 0.0001) but rose markedly higher in the sucrosomial^®^ Vit D3 group, with a statistically significant difference between the two groups (*p* = 0.02) i.e. sucrosomial^®^ Vit D3: from baseline mean ± SEM 11.6 ± 0.8 to 47.8 ± 5.3 ng/ml vs. SGC Vit D3: from baseline 12.6 ± 0.9 to 29.9 ± 2.9 ng/ml. At 6-weeks follow-up, circulatory 25(OH)D levels showed a further considerable increase in the sucrosomial^®^ Vit D3 treatment group to 66.1 ± 5.1 ng/ml (*p* = 0.049), with a statistically significant difference in the SGC Vit D3 group (38.2 ± 2.6 ng/ml) (*p* = 0.002), which showed only a minimal increase (*p* = 0.062). These results validate what was observed in study 1, *i.e*. they confirm an efficient absorption of sucrosomial^®^ Vit D3 as compared to the SGC Vit D3 supplement.

### 3.3. Safety analysis

Overall, in both studies, the Vit D3 treatment was well tolerated by all participants, and there were no treatment-related adverse effects, side effects, serious events, or discontinuation of the Vit D3 treatment due to safety reasons. There was no effect on the vital signs of any of the study participants. There were also no significant changes in the hematological and biochemical parameters studied, including calcium, liver enzymes, and creatinine (*p* ≥ 0.5) (see Supplementary Tables 1, 2 for study 1 and 2, respectively).

## 4. Discussion

**Figure 5:**
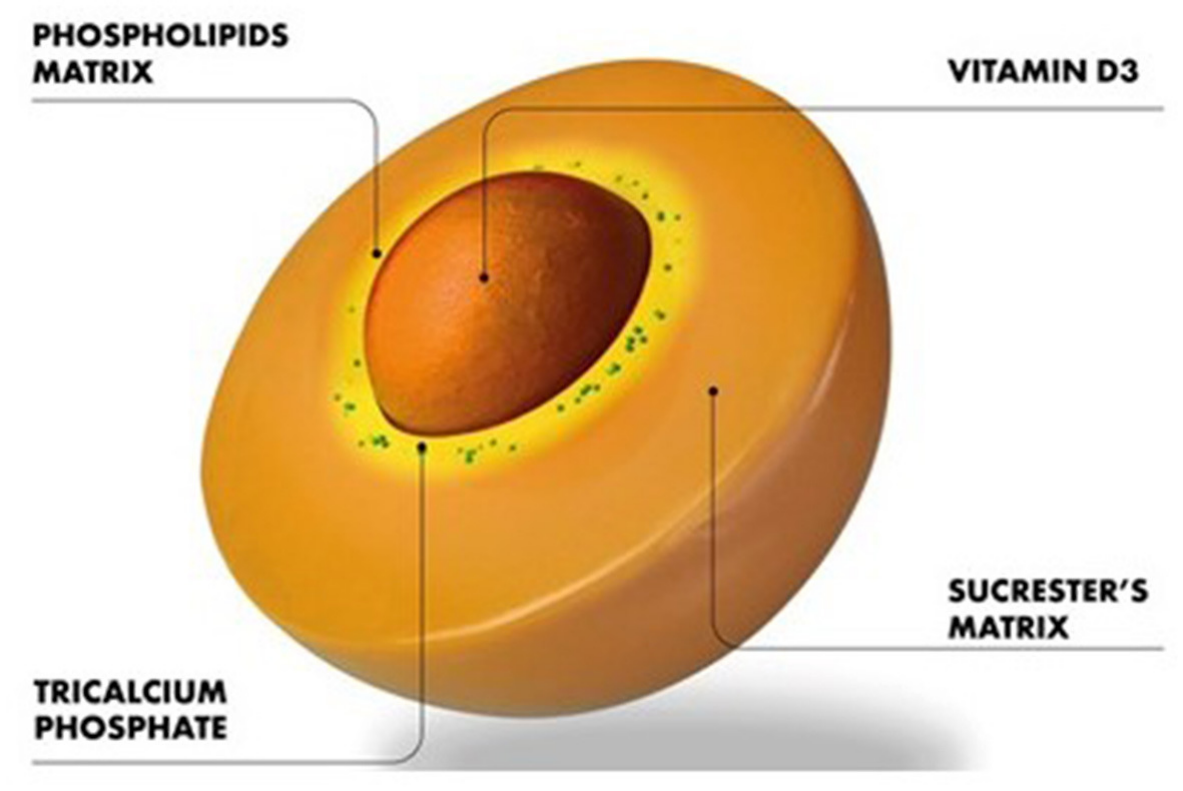
Structure of the sucrosomial^®^ biodelivery vehicle.

This exploratory prospective clinical trial for the first time assessed the efficacy of an innovative sucrosomial^®^ orodispersible Vit D3 formulation vs. a reference chewable tablet (study 1) and SGC (study 2) Vit D3 preparation in Vit D-deficient healthy adults and revealed that the sucrosomial^®^ Vit D3 is more efficacious in raising the circulating 25(OH)D levels owing to its efficient absorption. Moreover, the results of study 1 were validated by the results in study 2 which was conducted in a different province (Khyber Pakhtunkhwa) of Pakistan and involved participants of different ethnic background (Pashtuns) as compared to study 1 which was conducted in Balochistan province of Pakistan and involved participants of Baloch ethnicity, demonstrating the generalization of the results of efficient absorption/efficacy of sucrosomial^®^ Vit D3 supplementation. Vit D3 in the sucrosomial^®^ formulation is protected by a phospholipids, made mainly from sunflower lecithin plus a sucrester (sucrose ester) matrix (Figure 5). Additional stability and coating are achieved thanks to the presence of other ingredients as starch and tricalcium phosphate and forming a “sucrosome” delivery vehicle. The gastro-resistant properties of sucrosome (34) are believed to protect Vit D3 from degradation through its journey in the GI tract, decrease its interactions with food in the stomach, and promote transport and absorption across the intestinal epithelium to the circulatory system. The intact sucrosome allows the Vit D3 to reach the intestinal mucosa where it is absorbed as a vesicle-like structure through para-cellular and trans-cellular (M-cells) routes (29, 34–36). The unique structural, physicochemical, and pharmacokinetic properties make sucrosomial^®^ technology of significant interest in drug delivery applications. Reported evidence suggests that sucrester enhances the accumulation of drugs in CACO-2 cells (37) and acts as an enhances of intestinal permeability, as shown in animal model studies (38). In previous studies, with iron as an active agent, sucrosomial^®^ biodelivery vehicle has been shown to achieve high iron bioavailability and low gastrointestinal toxicity including in patients with chronic kidney disease, cancer, and bariatric surgery (29, 34, 36). In a recent study by Bertani et al. (39), an oral sucrosomial^®^ iron formulation was shown as effective as intravenous iron in treating anemia in patients with ulcerative colitis.

In addition to facilitating efficient intestinal absorption, another possible benefit of sucrosomial^®^ orodispersible Vit D3 could be its absorption in the oral cavity. Our study showed that the sucrosomial^®^ Vit D3 when taken inside the mouth, dissolves within moments when combined with saliva, and the fine micro-sized droplets containing Vit D3 are then possibly (and efficiently) absorbed through the buccal mucosa, palatal membranes, and sublingually into numerous capillaries and veins, close to the tissue surface. The oral cavity has a rich blood supply and is relatively permeable, facilitating an efficient route for Vit D absorption into the circulatory system. In the oral cavity route, Vit D3 bypasses the GI absorption pathway and is protected from degradation due to the pH and digestive enzymes of the GI tract (40), thus contributing to increased circulatory levels. It is speculated that the sucrosomial^®^ Vit D3 oral cavity absorption properties may well prove superior for those with GI malabsorption syndromes such as individuals with cystic fibrosis (41), Crohn's disease (42), intestinal reresection (43, 44), ulcerative colitis, and liver disease (45, 46), who fail to achieve adequate circulatory 25(OH)D levels, despite routine supplementation and for individuals with difficulty swallowing such as the elderly, young children, and babies.

The absorption of Vit D3 preparations in the oral cavity has been investigated in several studies. In study by Khazi et al. involving cystic fibrosis patients, a greater bioavailability of powder Vit D preparation was observed compared with an oil-soluble vehicle (22). In study by Satia et al., a buccal spray Vit D3 preparation led to significantly higher circulatory 25(OH)D concentration as compared with SGC Vit D3 preparation, in both healthy subjects and patients with malabsorption syndrome (26). In study by Cupone et al., an orodisintegrable film Vit D3 preparation showed disintegration of the vehicle in less than 1 min and the Vit D3 release was ≥75% after 15 min (20). In a similar study by Radicioni et al., a higher Vit D3 bioavailability was achieved with an orodispersible Vit D3 preparation compared with an oral solution preparation in healthy subjects (25). In general, among most of the previously reported studies of Vit D3 oral cavity absorption, the sucrosomial^®^ Vit D3 preparation appears to be the most effective for achieving increased bioavailability of Vit D, owing to its unique structural and physiochemical characteristics. However, further studies are required to establish the absorption of sucrosomial^®^ Vit D3 in the oral cavity.

This study also demonstrated excellent safety and tolerability profile of the sucrosomial^®^ Vit D3 supplement in high dosage, and no cases of hypercalcemia occurred. There were no negative effects on the liver function enzymes and kidney function (creatinine) as well as hematology in any of the study participants throughout the study 6-weeks period. The adequately higher circulatory Vit D levels achieved with high-dose sucrosomial^®^ Vit D3 supplementation in this study, alongside excellent safety and tolerability, could provide a rationale for clinical trials to assess whether such Vit D levels are associated with immunomodulatory pharmacological effects in autoimmune diseases and infection risk (47–49).

Our study is not free from limitations. The relatively small sample size, open-label nature, and absence of diseased participants such as frail elderly, those with chronic conditions, e.g. chronic liver or kidney diseases, and malabsorption syndrome are some of the drawbacks of this study. Nevertheless, those involved in the randomization, enrolment of participants, drawing the participants' blood samples, clinical diagnostic laboratories evaluating the serum 25(OH)D levels, and investigators assessing the outcomes were blinded to the intervention. Moreover, the results of study 1 were validated by results of the study 2 conducted in a different geographic location and involving participants of different ethnic background, demonstrating the wider applicability of the results.

## 5. Conclusion

According to the results of our study, the sucrosomial^®^ orodispersible Vit D3 preparation dissolves and absorbs efficiently in the GI system, leading to adequately higher and sustained circulatory Vit D levels as compared to a chewable tablet and soft gel capsule forms of Vit D3 preparations in Vit D-deficient healthy adults, and thus could contribute effectively to body protection against diseases associated with VDD.

## Data availability statement

Data supporting the findings of this study are included within the article and/or Supplementary material, further inquiries can be directed to the corresponding author.

## Ethics statement

The study was reviewed and approved by the BMCH Ethical Board Committee (Ref. no. BMCH/EBC/5500) and LRH Institutional Review Board (Ref. no. 696/LRH/MTI). The patients/participants provided their written informed consent to participate in this study.

## Author contributions

AK, AB, AAB, and SAb: conceptualization, methodology, resources, supervision, and project administration. AB, SAb, AAB, FA, MB, SM, and HZ: investigation and data curation. MF and GC: statistical analysis. AK: writing—original draft preparation, conception and coordination of the study, full access to all data in the study, and responsibility for the integrity of the data and the accuracy of the data analysis. ID, SAs, EB, GT, AG, ST, and SK: writing—reviewing and editing. All authors have read and approved the final version of the manuscript for submission.
