# Supplementary material for: A comparative absorption study of sucrosomial® orodispersible vitamin D3 supplementation vs. a reference chewable tablet and soft gel capsule vitamin D3 in improving circulatory 25(OH)D levels in healthy adults with vitamin D deficiency—Results from a prospective randomized clinical trial
Source: Front Nutr. 2023 Aug 17;10:1221685. doi: 10.3389/fnut.2023.1221685 (PMC10469777; doi:10.3389/fnut.2023.1221685)
Supplement: Supplementary file 1 [file Table_1.pdf]

Supplementary Table 1. Biochemistry and hematology of the participants before and after Vit D3 supplementation in study 1. Data are presented as mean ± SEM. No statistically significant differences over time were observed within each Vit D3 treatment group (Wilcoxon test, $p = 1$).

**Parameter**	**Sucrosomial® Vit D3 treatment group (n=10)**		**Chewable tablet Vit D3 treatment group (n=12)**	
	**Baseline**	**After six weeks**	**Baseline**	**After six weeks**
Calcium (mg/dL)	9.8 ± 0.0	9.4 ± 0.2	9.9 ± 0.1	9.8 ± 0.0
Creatinine (mg/dL)	0.9 ± 0.0	0.9 ± 0.0	0.8 ± 0.0	12.3 ± 7.7
**Liver enzymes**				
Total bilirubin (mg/dL)	0.4 ± 0.0	0.5 ± 0.0	0.3 ± 0.0	0.4 ± 0.0
SGPT (ALT) (U/L)	40.5 ± 8.7	34.6 ± 5.6	25.1 ± 3.2	19.8 ± 3.1
ALP (U/L)	76.7 ± 5.4	89.3 ± 11.8	80.0 ± 5.5	92.5 ± 7.4
GGT (U/L)	28.9 ± 4.4	30.4 ± 5.5	39.9 ± 17.0	56.2 ± 34.4
SGOT (AST) (U/L)	30.7 ± 4.2	29.3 ± 3.0	28.5 ± 3.4	26.0 ± 3.6
**Hematology**				
Haemoglobin (g/dL)	16.2 ± 0.6	16.2 ± 0.6	13.5 ± 0.8	13.5 ± 0.8
RBCs (mil/µL)	5.5 ± 0.2	5.5 ± 0.2	5.2 ± 0.2	5.2 ± 0.2
Haematocrit (%)	49.1 ± 1.9	47.1 ± 1.6	42.8 ± 2.0	41.6 ± 2.3
MCV (fl)	88.3 ± 2.1	85.4 ± 2.0	81.8 ± 3.2	79.5 ± 3.3
MCH (pg)	29.2 ± 0.6	29.4 ± 0.6	25.7 ± 1.3	25.8 ± 1.3
MCHC (g/dL)	33.1 ± 0.5	34.4 ± 0.3	31.36 ± 0.65	32.4 ± 0.5
TLC (/ccm)	7492.0 ± 540.5	7379.0 ± 366.2	7084.1 ± 539.2	6425.0 ± 418.8
Neutrophils (%)	57.0 ± 1.9	56.2 ± 2.1	52.5 ± 1.9	51.9 ± 1.6
Lymphocytes (%)	33.2 ± 1.6	36.5 ± 3.3	38.0 ± 1.8	38.1 ± 1.9
Eosinophils (%)	2.6 ± 0.5	3.3 ± 1.0	1.9 ± 0.3	2.6 ± 0.7
Monocytes (%)	7.2 ± 0.5	7.0 ± 0.6	7.5 ± 0.7	7.2 ± 0.5
Platelets (/ccm)	246000.0 ± 20149.4	249100.0 ± 18881.1	324583.3 ± 16214.6	290750.0 ± 13835.6

ALP, alkaline phosphatase; GGT, gamma-glutamyl transferase; MCH, mean corpuscular haemoglobin; MCHC, mean corpuscular haemoglobin concentration; MCV, mean corpuscular volume; RBCs, red blood cells; SGOT (AST), serum glutamic-oxaloacetic transaminase (aspartate aminotransferase); SGPT (ALT), serum glutamic-pyruvic transaminase (alanine aminotransferase); TLC, total leukocyte count.
