# Supplementary material for: A comparative absorption study of sucrosomial® orodispersible vitamin D3 supplementation vs. a reference chewable tablet and soft gel capsule vitamin D3 in improving circulatory 25(OH)D levels in healthy adults with vitamin D deficiency—Results from a prospective randomized clinical trial
Source: Front Nutr. 2023 Aug 17;10:1221685. doi: 10.3389/fnut.2023.1221685 (PMC10469777; doi:10.3389/fnut.2023.1221685)
Supplement: Supplementary file 2 [file Table_2.pdf]

Supplementary Table 2. Biochemistry and hematology of the participants before and after Vit D3 supplementation in study 2. Data are presented as mean ± SEM. No statistically significant differences over time were observed within each Vit D3 treatment group (Wilcoxon test, $p \geq 0.5$).

Parameter	Sucrosomial® Vit D3 treatment group (n=6)		Soft gel capsule Vit D3 treatment group (n=8)	
	Baseline	After six weeks	Baseline	After six weeks
Calcium (mg/dL)	9.6 ± 0.2	9.6 ± 0.1	9.7 ± 0.2	9.7 ± 0.2
Creatinine (mg/dL)	0.7 ± 0.0	0.6 ± 0.0	0.8 ± 0.0	0.7 ± 0.1
**Liver enzymes**				
Total bilirubin (mg/dL)	0.6 ± 0.0	0.5 ± 0.1	0.5 ± 0.0	0.5 ± 0.1
SGPT (ALT) (U/L)	23.1 ± 3.5	15.3 ± 0.9	24.5 ± 3.4	22.8 ± 3.9
ALP (U/L)	148.0 ± 10.2	148.1 ± 8.8	111.8 ± 15.4	138.2 ± 13.7
**Haematology**				
Haemoglobin (g/dL)	12.7 ± 0.2	12.5 ± 0.2	12.6 ± 0.6	12.6 ± 0.6
RBCs (mil/µL)	4.4 ± 0.1	3.9 ± 0.3	4.7 ± 0.1	4.8 ± 0.1
MCV (fl)	78.0 ± 9.1	77.4 ± 8.9	82.1 ± 2.7	81.0 ± 2.6
MCH (pg)	29.1 ± 0.8	29.2 ± 0.7	26.9 ± 0.9	26.8 ± 1.1
MCHC (g/dL)	33.7 ± 0.3	34.1 ± 0.2	32.8 ± 0.5	31.7 ± 1.3
TLC (/ccm)	7448.3 ± 582.8	7390.0 ± 724.0	8572.5 ± 736.7	7801.2 ± 525.4
Neutrophils (%)	56.8 ± 2.0	59.5 ± 1.8	60.0 ± 0.7	57.5 ± 2.0
Lymphocytes (%)	33.0 ± 2.4	30.1 ± 2.5	29.7 ± 1.1	33.8 ± 2.3
Eosinophils (%)	3.8 ± 1.0	4.1 ± 1.2	2.8 ± 0.3	2.0 ± 0.2
Monocytes (%)	6.3 ± 0.5	6.1 ± 0.4	7.3 ± 0.8	6.6 ± 0.5
Platelets (/ccm)	268833.3 ± 20445.7	268833.3 ± 28193.8	274000.0 ± 18198.3	268125.0 ± 21962.1

ALP, alkaline phosphatase; MCH, mean corpuscular haemoglobin; MCHC, mean corpuscular haemoglobin concentration; MCV, mean corpuscular volume; RBCs, red blood cells; SGPT (ALT), serum glutamic-pyruvic transaminase (alanine aminotransferase); TLC, total leukocyte count.
